# Effect of foliar spray selenium on antioxidant defense system, yields, fatty acid composition, and mineral concentrations in flax (*Linum usitatissimum* L.)

**DOI:** 10.3389/fpls.2025.1600173

**Published:** 2025-06-13

**Authors:** Yaping Xie, Yangchen Zhou, Limin Wang, Wenjuan Li, Wei Zhao, Zhao Dang, Xingzhen Wang, Yanqiao Duan, Jianping Zhang, Minlu Yuan, Gang Wang

**Affiliations:** ^1^ Crop Research Institute, Gansu Academy of Agricultural Sciences, Lanzhou, China; ^2^ College of Agronomy, Gansu Agricultural University, Lanzhou, China

**Keywords:** flax, selenium, antioxidant, productivity, quality, biofortification

## Abstract

**Introduction:**

Flax is an important multipurpose oil crop with high-quality seed, oil, and fibre.

**Methods:**

This study aims to investigate the effect of selenium (Se) fertilization on the flax antioxidant defense system, yields, fatty acid composition, and mineral concentrations. A field experiment was conducted in Gansu, Northwest China in 2022, 2023, and 2024, by foliar application of Se at early budding, early flowering, and early seed-filling using a low-pressure hand sprayer. A randomized complete block design with three replicates was employed by analyzing physiological and biochemical characteristics to assess yield and quality of flax.

**Results:**

Result showed that harvest year significantly influenced proline, soluble sugar, oil, oleic, linoleic and linolenic acid contents, the superoxide dismutase activity in leaves, lignans, Se, calcium, and iron concentrations in seeds, the number of capsules per plant, and the yields of seed, lignans, oil, and oil cake. The proline and soluble sugar contents, superoxide dismutase, peroxidase, and catalase activities and number of capsules per plant, Se, zinc, and iron concentrations, as well as seed, oil, lignans, and flaxseed oil cake yields increased by foliar Se, while reducing malondialdehyde and cadmium levels, compared to without Se application. Specifically, the highest number of capsules per plant was observed at Se of 30 mg L^–1^; the yields of seed, oil, lignans, and flaxseed oil cake were achieved at Se of 40 mg L^–1^, however, there was no significant difference in foliar Se between 30 and 40 mg L^–1^ across harvests. Moreover, the iron and zinc concentrations reached the peaked at Se of 20 mg L^–1^, and Se concentration was measured at 50 mg L^–1^. In conclusion, foliar application of appropriate Se is an effective agronomic management approach to enhance yields and quality of flax by enhancing the antioxidant defense as well as promoting absorption and accumulation of Se, iron, and zinc in seeds.

**Discussion:**

Future experiments will systematically investigate the effects of Se type, foliar spray timing, and application frequency on the nutrient content, hormone levels, stress resistance, yield, and quality of flax.

## Introduction

Flax (*Linum usitatissimum* L.), known as linseed, is an ancient vital and versatile crop with multiple functions and purposes. It is cultivated in many parts of world for its fiber, oil and industrial applications (Zuk et al., 2015). Flaxseed is renowned for its diverse bioactivities, including anti-atherogenic, anti-thrombotic, anti-arrhythmic, and anti-inflammatory effects, which are attributed to its rich content of α-linolenic acid, lignans, dietary fiber, proteins, and minerals (Mueed et al., 2022). These components endow flaxseed with antioxidant and functional properties that benefit human health (Parikh et al., 2019; Suri et al., 2020). Whole flaxseed flour, with excellent functional and nutritional attributes, can be utilized to enrich foods, meeting the requirements of human meals while promoting health and preventing diseases (Zhang et al., 2023). Flax oil extracted from flaxseed is not only an important high-quality edible vegetable oil (Xie et al., 2020) but also serves as a raw material for biodiesel production due to its environmentally friendly, renewable, and sustainable properties (Perera et al., 2025).Additionally, it is used in the production of ethyl esters (Juszczyk et al., 2019). Flaxseed oil cake, generated as a by-product of cold-press production from flaxseed, is a valuable resource (Salachna et al., 2024). This cake serves as an excellent source of dietary fiber, proteins, minerals (Mg, Ca, Zn, Fe), carbohydrates, bioactive chemicals, vitamins and antioxidatives (Kaur et al., 2021). It can be utilized as a potential ingredient in healthy food products for humans (Kaur et al., 2022), as a component of animal feed, solid compost, or organic fertilizers (Mannucci et al., 2019; Salachna et al., 2024). Furthermore, flax shives, which are the residual material after processing flax fiber from the stem, have been used for soil bioremediation (Dey et al., 2021), wastewater treatment (Dey et al., 2021), and as adsorbents, composites, fuels and chemicals (Perera et al., 2025; Zhang et al., 2025; Tan et al., 2025). Nowadays, there is a dramatically increasing demand for flaxseed in China. Additionally, flax’s productive capability is comparatively lower than that of other oil crops such as rapeseed, soybean, and groundnut, which are more familiar to farmers. Therefore, it is both urgent and important to enhance the productive capability of flax under these circumstances.

Selenium (Se) is an essential micronutrient for maintaining human health. Its functions include bolstering the immune system, reducing the risk of cardiovascular disease, regulating thyroid function, detoxification, anticancer effects, and antiviral activity (Silva et al., 2023). Humans primarily acquire Se through their daily diet and/or nutritional supplements (Yuan et al., 2023). Selenium deficiency can lead to various diseases, while excessive Se can also be harmful to human health (Zhang and Song, 2021). In China, approximately two-thirds of the population is at risk of Se deficiency. Dietary Se fortification can be achieved through the biofortification of edible crops via foliar spraying or soil application to enhance its levels in the edible parts of plants (Sarwar et al., 2020). Hence, Se biofortification of crops has been identified as one of the most efficient methods to combat Se deficiency (Avnee et al., 2023).

Previous studies have highlighted the crucial role of Se in various metabolic activities that promote plant development in higher plants (Lyons et al., 2009) and enhance yield and quality (Zhang et al., 2023). Numerous studies have demonstrated the beneficial effect of Se on plants growth and development by improving SOD, POD, and CAT activities (Dai et al., 2019; Shalaby et al., 2017; Hussein et al., 2019), declining MDA content (Logvinenko et al., 2022; Haghighi et al., 2019), and increasing soluble sugar and proline content (Azimi et al., 2021; Wu et al., 2023), resulting in enhancing antioxidant defense system under various abiotic stress. For example, Se has been shown to improve growth under high temperature stress of sorghum (*Sorghum bicolor* L*.Moench*) (Djanaguiraman et al., 2010) and pepper (*Piper nigrum* L.) (Haghighi et al., 2019), tomato (*Solanum lycopersicum* L.) (Alves et al., 2020) and *Dracocephalum moldavica* L. (Azimi et al., 2021) growth under cadmium (Cd) stress, wheat (*Triticum aestivum* L.) (Nawaz et al., 2015) and camelina (*Camelina sativa* L) and canola (*Brassica napus* L.) (Ahmad et al., 2021) under drought stress, tea (*Camellia sinensis* L.) under glufosinate stress (Yu et al., 2024), kale (*Brassica oleracea* var. *sabellica*) under microplastics pollution (Tong et al., 2024), and tomato under salt stress (Wu et al., 2023). Furthermore, many studies have noted the role of Se in significantly decreasing MDA levels in *Artemisia annua* L (Logvinenko et al., 2022), alfalfa (*Medicago sativa* L.) (Bai et al., 2019), tomato (Wu et al., 2023), *Dracocephalum moldavica* L (Azimi et al., 2021), and rice (*Oryza sativa* L.) (Lin et al., 2012). Additionally, Se has been reported to significantly increase proline content in Dracocephalum moldavica L (Azimi et al., 2021), maize (*Zea mays* L.) (Sharma et al., 2018), Chinese cabbage (Dai et al., 2019), and tomato (Alves et al., 2020), as well as regulate soluble sugar in tomato (Wu et al., 2023), groundnut (*Arachis hypogaea* L.) (Hussein et al., 2019), and tea (Li et al., 2021).

The application of Se to enhance crop yield and Se content in seed/grain has been documented in various crops, including lentil (*Lens culinaris* L.) (Ekanayake et al., 2015), oilseed rape (*Brassica napus*) (Lyons et al., 2009), canola (Ahmad et al., 2021), camelina (Ahmad et al., 2021), soybean (*Glycine max* L.) (Djanaguiraman et al., 2005), sorghum (Djanaguiraman et al., 2010), safflower (*Carthamus tinctorius* L.) (Sher et al., 2022), and wheat (Nawaz et al., 2015; Liu et al., 2021). However, Wang et al. (2013) reported that foliar Se applications had no significant effect on grain yield in maize, although the Se concentration in the grain increased markedly. Meanwhile, there is limited research investigating the impact of Se on lignans concentration. Furthermore, few studies have comprehensively explored the influence of Se on oil content and fatty acids composition. Notably, Davoudi et al. (2019) examined the effect of foliar Se application on oil content and fatty acid composition in rapeseed (*Brassica napus* L.). Their findings revealed that Se application significantly increased oil levels and altered fatty acid composition.

The potential interactions and competition relationships between Se and other major and trace elements present a critical scientific issue. Numerous studies have reported on the influence of Se on the concentrations of iron (Fe), zinc (Zn), magnesium (Mg), calcium (Ca), and Cd in the edible portions of various crops (Liu et al., 2021; Bai et al., 2019; Huang et al., 2018). Bai et al. (2019) found that Se application promoted Fe and Ca uptake in alfalfa. In rice, Li et al. (2019) reported that appropriate level of Se increased the levels of Fe, Zn, and Se in grains. Similar effects was observed in black-grained wheat, where Se application increased the levels of Fe, Zn, and Se (Liu et al., 2021). Literature also confirms that Se application maximizes Mg accumulation in wheat grains under both normal and water-deficit conditions (Nawaz et al., 2015). Cadmium in crop edible parts, posing serious health risks to humans (Hamid et al., 2019). An ideal crop for human consumption should be rich in essential nutrients such as Se while minimizing the accumulation of toxic elements like Cd. Se application is considered a promising strategy to reduce Cd accumulation in plants (Zhou et al., 2020). Recent studies have demonstrated a significant antagonistic effect of Se on Cd accumulation in various crops (Affholder et al., 2019; Alves et al., 2020). For instance, Huang et al. (2018) showed that exogenous selenite and selenate treatments significantly decreased Cd in rice by 36.5% and 25.3%, respectively, compared to control treatments.

Limited information is available in the existing literature regarding the effects of Se on seed yield, lignans concentration, oil content and yield, fatty acid profile, and the concentrations of Se, Ca, Mg, Fe, Zn, and Cd in seeds and flaxseed oil cake. We hypothesized Se could enhance antioxidant defense, improve productivity and quality of flax. Therefore, the objective of this study was to determine the optimal concentration of foliar-applied Se to modulate seed and oil yields, lignans levels, fatty acid composition, and mineral element concentrations in flaxseed, as well as the yield of flaxseed oil cake.

## Materials and methods

### Experimental design and field management

**Figure 1:**
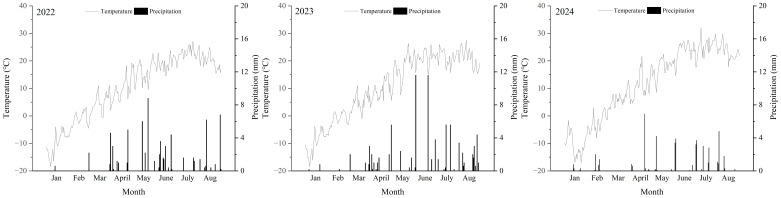
Mean air temperatures and percipitation in 2022, 2023, and 2024 harvests in Qingwangchuan, China.

The experiment was conducted at the Qinwangchuan Modern Agricultural Comprehensive Experimental Station, Gansu Academy of Agricultural Sciences (103°41′E, 36°35′N, and altitude of 1890 m) in 2022, 2023, and 2024. Wheat was the previous crop in three harvests. During the growing season from March to August, monthly temperatures ranged from 2°C to 32°C, with the lowest temperature recorded in April and the highest in August. Total precipitation varied between 258 mm and 316 mm during this period across the three harvests (**Figure 1**).

The experiment was performed with a randomized complete block design with three replicates. Each plot size was 2 by 3 m. Flax cultivar Longya 13 was sown on 8 April, 2022; 8 April, 2023; and 12 April, 2024, at a seeding rate of 1050 viable seeds m^–2^ to achieve a target density of 750 plants m^–2^. The treatments consisted of foliar spray Se concentrations of 0, 20, 30, 40, and 50 mg L^–1^ Se in the form of organic Se, these treatments were designated as Se0 [the zero Se control (CK)], Se20, Se30, Se40, and Se50, respectively; which were prepared with distilled deionized water. The Se solution was applied via foliar spray at early budding, early flowering, and early seed-filling stages by a low-pressure hand sprayer, applying 50 mL m^–2^. Nitrogen (N) fertilization was provided at a rate of 100 kg N ha^–1^ as urea; with 70% applied as basal fertilizer and 30% applied at the budding stage during irrigation. Phosphorus (P) was supplied at a rate of 80 kg P_2_O_5_ ha^-^¹ as calcium superphosphate, and potassium (K) was applied at 40 kg K_2_O ha^-^¹ as potassium sulfate, both as basal fertilizers.

Harvesting was accomplish on 18 August, 2022; 20 August, 2023; and 20 August, 2024.

### Soil and plant samplings

Soil samples were collected from a depth of 0–30 cm prior to sowing in each harvest and analyzed for chemical characteristics (**Supplementary Table 1**). The soil is classified as Arenosols (FAO, 2015). The pH was measured using potentiometry, soil organic matter content was determined by potassium dichromate volumetry, P and N concentrations were analyzed by the Colorimetric Molybdenum-Blue method and the micro-Kjeldahl method, respectively (Xie et al., 2023). The total Se concentration in the soil was quantified using an inductively coupled plasma optical emission spectrometer (ICP-MS, Agilent 7900, Palo Alto, California, USA).

Samples of 30 plants were collected from all treatments five days after Se foliar application during the early seed-filling stage. Plants were sampled from the two central rows of each plot and separated into leaves and other parts. Leaf samples were detached, washed, and frozen in liquid nitrogen at –80°C to analyze SOD, POD, and CAT activities, as well as MDA, proline, and soluble sugar contents (2023 and 2024) (A degradation of leaf samples occurred in the refrigerator due to a power failure in 2022). At maturity, a 1-m length of plant rows was randomly selected from the two central rows of each plot to record the number of capsules per plant and the number of seeds per capsule (2023 and 2024). On the day of harvest, crops from each plot were hand-harvested separately using a sickle, and seed yield was measured.

### Malondialdehyde, proline, and soluble sugar

Malondialdehyde (MDA) content was determined using thiobarbituric acid reacting substances (TBARS) as described by Djanaguiraman et al. (2010). In brief, a 0.1 mg frozen leaf sample was homogenized in 5 mL 0.1% trichloroacetic acid (TCA). The homogenate was centrifuged at 10,000 g for 5 min at 4°C. Subsequently, 0.3 mL of the supernatant was mixed with 1.2 mL 0.5% thiobarbituric acid (TBA) prepared in 20% TCA and incubated at 95°C for 30 min. After cooling the samples for 5 min, they were centrifuged again at 10,000 g for 10 min at 25°C. Absorbance was measured at 532 nm using a Hitachi U-2000 double-beam UV/Vis spectrophotometer (Hitachi, Lake Sherwood, MO, USA). Malondialdehyde (MDA) concentration was expressed in nmol g^–1^ of fresh weight.

Proline content was confirmed using fresh leaves (0.5 g) were homogenized in 3% sulphosalicylic acid and filtered. The mixture filtrate was added with 1 mL each of acid ninhydrin and glacial acetic acid and was placed in boiling water for 1 h. Toluene (4 mL) was added to the mixture, the absorbance was measured spectrophotometrically at 520 nm and converted to µmol g^−1^ fresh weight against standard proline (Azimi et al., 2021).

Soluble sugar concentration was measured using anthrone colorimetry (Lu et al., 2024). Frozen leaf sample (0.1 mg) was homogenized, transferred into a graduated glass test tube with a stopper, 5 ml distilled water was added, extracted for 30 minutes (twice). The extract is filtered into a 25ml volumetric bottle, rinse test tube repeatedly. To this, 1 mL distilled water and 4 mL 0.2% anthrone solution were added. The mixture was thoroughly shaken, heated for 15 min. Following that, the tube was removed and allowed to cool to 25°C. Soluble sugar content was determined colorimetrically at 620 nm using a ultraviolet spectrophotometer (Thermo FisherScientific Inc., Waltham, MA, USA).

### Superoxide dismutase, peroxidase, and catalase

Frozen sample (0.5 g) was extracted in 10 mL sodium phosphate buffer (0.1 mol L^−1^) containing polyvinylpolypyrrolidone (2%, w/v). The extracted solution pH for SOD and POD were 6.8 and 6.4, respectively. The homogenate was centrifuged at 12,000 × g for 30 min at 4°C. SOD reaction solution included of 0.05 mol L^−1^ sodium phosphate buffer (1.7 mL), 0.014 mol L^−1^ methionine (0.3 mL), 0.75 mmol L^−1^ inhibition of nitroblue tetrazolium (NBT) (0.3 mL), 1.0 μmol L^−1^ EDTA (0.3 mL), 20.0 μmol L^−1^ riboflavin (0.3 mL), and enzyme extract (0.1 mL). SOD activity was measured by monitoring 50% NBT photochemical reduction. The solution was observed at 560 nm, and the result was shown as U g^−1^ FW (Li et al., 2021; Yu et al., 2024).

POD reaction solution comprised 0.05 mol L^−1^ sodium phosphate buffer (2.7 mL), of 0.02 mol L^−1^ H_2_O_2_ (0.1 mL), 0.02 mol L^−1^ guaiacol (0.1 mL) as a substrate, and 0.1 mL of enzyme extract. The solution was analyzed at 470 nm, and the result was shown as U g^−1^ FW (Yu et al., 2024). The reaction mixture for the assay of CAT activity contained sodium phosphate buffer (pH 7.5), enzyme extract, H_2_O_2_ and the activity was recorded as change in absorbance at 240 nm for 3 min at an interval of 30 s (Sharma et al., 2018).

### Oil content and yield

The oil content was measured using the Soxhlet extraction method as specified by American Oil Chemists’ Society (1983). The oil yield was assessed following the methodology outlined by Xie et al. (2020), as follow:


(1) $$\text{Oil yield }({\text{kg ha}}^{–1})=\text{oil content}\;(\%)\times \text{seed yield }({\text{kg ha}}^{–1})$$

### Lignans concentration and yield, and fatty acid composition in seeds

Lignans concentration and fatty acid composition in seeds of flax were quantified based on percent dry matter (Wilcox and Shibles, 2001) using a near-infrared reflectance diode array analyzer (Perten Instruments, Stockholm, Sweden), as detailed in previous literature (Xie et al., 2022). Calibrations models were developed using Thermo Galactic Grams PLS IQ software (Perten Instruments, Stockholm, Sweden). The calibration curve was annually updated on the basis of independent samples analyzed by high-performance liquid chromatography (Xie et al., 2020).

Lignans yield was estimated as follow:


(2) $$\begin{array}{c} \text{Lignans yield }({\text{kg ha}}^{–1})\\ =\text{lgnans concentration }({\text{mg g}}^{–1})\times \text{seed yield }({\text{kg ha}}^{–1}) \end{array}$$

### Concentration of Se, Zn, Fe, Ca, Mg, and Cd in seeds

Seed samples (0.25 g) were weighed and digested for approximately 2h at 110–120°C by concentrated HNO_3_ and hydrogen peroxide in calibrated 50 ml tubes. Followed the samples are brought to 25 ml total volume with deionized water and then analyzed by an inductively coupled plasma mass spectrometer (Agilent 7900, Agilent Technologies, Palo Alto, California, USA). The concentrations of Se, Fe, Zn, Mg, Ca, and Cd in seeds were detected. Details of the procedures are described in a previous article (Xie et al., 2022).

### Flaxseed oil cake

Oil cake of flaxseed was calculated as follow:


(3) $$\begin{array}{c} \text{Flaxseed oil cake }({\text{kg ha}}^{–1})\\ =\text{seed yield }({\text{kg ha}}^{–1})–\text{oil yield }({\text{kg ha}}^{–1}) \end{array}$$

### Data analysis

The data were subjected to analysis of variance (ANOVA) using SPSS (version 19, Inc., Chicago, IL, USA). Year was termed “harvest” for ANOVA and considered a random effect while Se rate was fixed effect. Means were compared using the Tukey test at a significance level of 0.05. All results are presented as means ± standard error (SE) (n = 3).

## Results

### Malondialdehyde, proline, and soluble sugar

**Figure 2:**
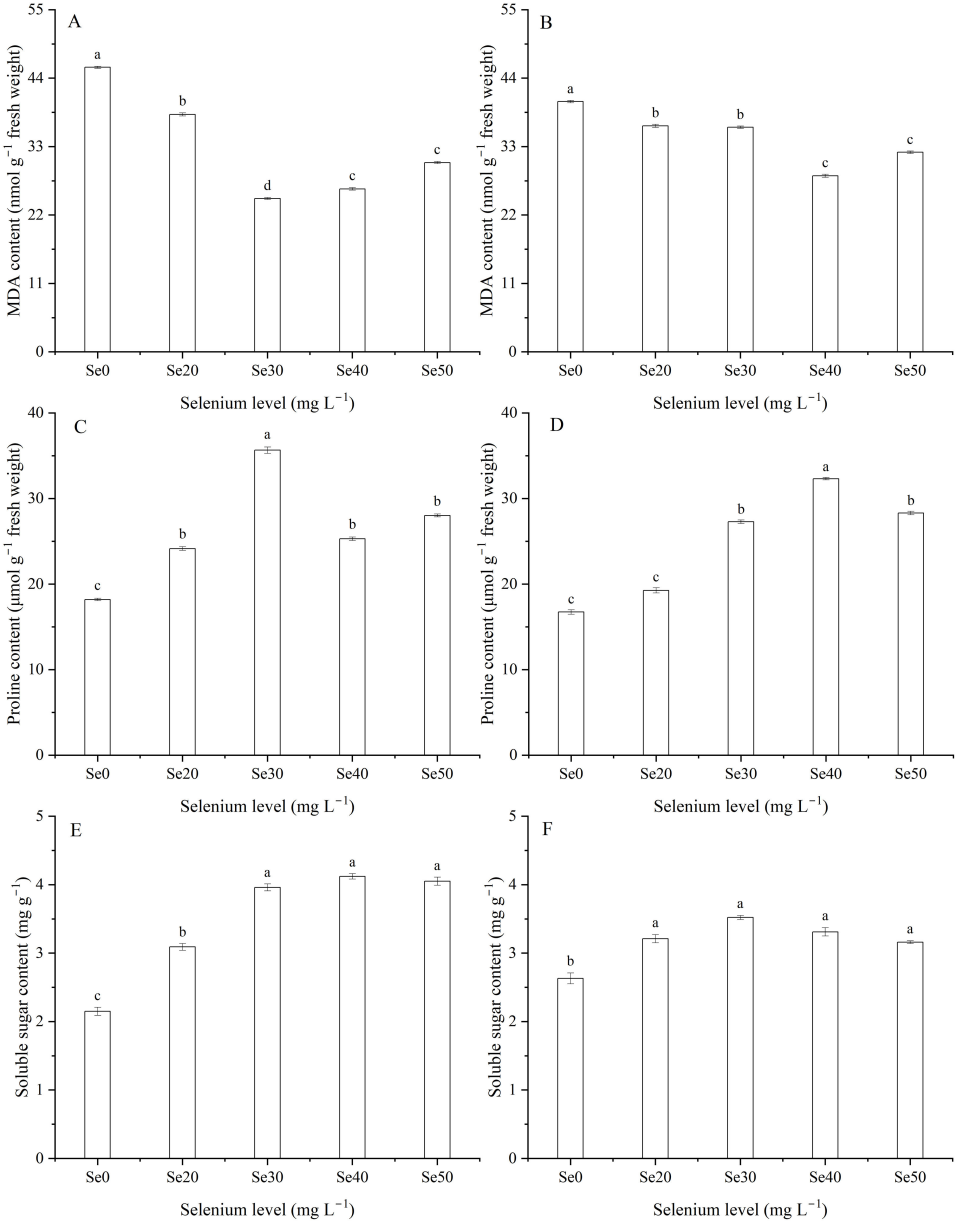
Effect of foliar selenium (Se) application on Malondialdehyde (MDA) **(A)** (2023) and **(B)** (2024), proline **(C)** (2023) and **(D)** (2024), and soluble sugar **(E)** (2023) and **(F)** (2024) contents in flax leaves. Different letters indicate means in the same harvest that are significantly different at *P* = 0.05 according to Tukey’s test. Vertical bars represent standard errors (n=3).

Selenium level and the interaction between harvest and Se significantly decreased MDA content (**Table 1**; **Figures 2A, B**). Compared with the control, the MDA content in leaves decline by an average of 34.7 and 17.5% with spray Se treatments in 2023 and 2024, respectively. The lowest levels were observed at Se30 and Se40 in 2023 and 2024, respectively. Conversely, foliar Se at different doses led to varying degrees of proline accumulation in leaves (**Table 1**; **Figures 2C, D**). Specifically, compared to the control, proline content increased by an average of 55.3 and 60.1% in 2023 and 2024, respectively. The highest proline levels were recorded at Se30 in 2023 and Se40 in 2024. Similarly, foliar Se application resulted in a significant increase in soluble sugar content, with varying amplitudes (**Table 1**; **Figures 2E, F**). The soluble sugar content improved by an average of 70.0 and 25.5% in 2023 and 2024 than the controls, respectively. The maximum soluble sugar contents were recorded at Se40 and Se30 in 2023 and 2024, respectively.

**Table 1:** Analysis of variance results for dependent variables.

Dependent variable	Harvest	Selenium (Se) rate	Harvest×Se rate
Malondialdehyde (MDA)	ns	**	*
Proline	*	**	*
Soluble sugar	*	**	*
Superoxide dismutase (SOD)	*	*	*
Peroxidase (POD)	ns	**	*
Catalase (CAT)	ns	*	*
Number of capsules per plant	**	*	**
Number of seeds per capsule	ns	ns	ns
1000-seed weight	ns	ns	*
Seed yield	**	**	*
Oil content	*	ns	ns
Oil yield	**	**	**
Lignans concentration	*	ns	*
Lignans yield	*	**	**
Stearic acid	ns	ns	ns
Palmitic acid	ns	ns	ns
Oleic acid	*	ns	*
Linoleic acid	**	ns	ns
Linolenic acid	*	ns	*
Selenium concentration in seed	**	**	ns
Calcium concentration in seed	**	ns	ns
Magnesium concentration in seed	ns	ns	ns
Iron concentration in seed	*	**	ns
Zinc concentration in seed	ns	*	ns
Cadmium concentration in seed	ns	**	ns
Flaxseed oil cake yield	*	**	ns

* Significant at *P* < 0.05. ** Significant at *P* < 0.01. ns indicates no significant difference at *P* = 0.05.

### Superoxide dismutase, peroxidase, and catalase

**Figure 3:**
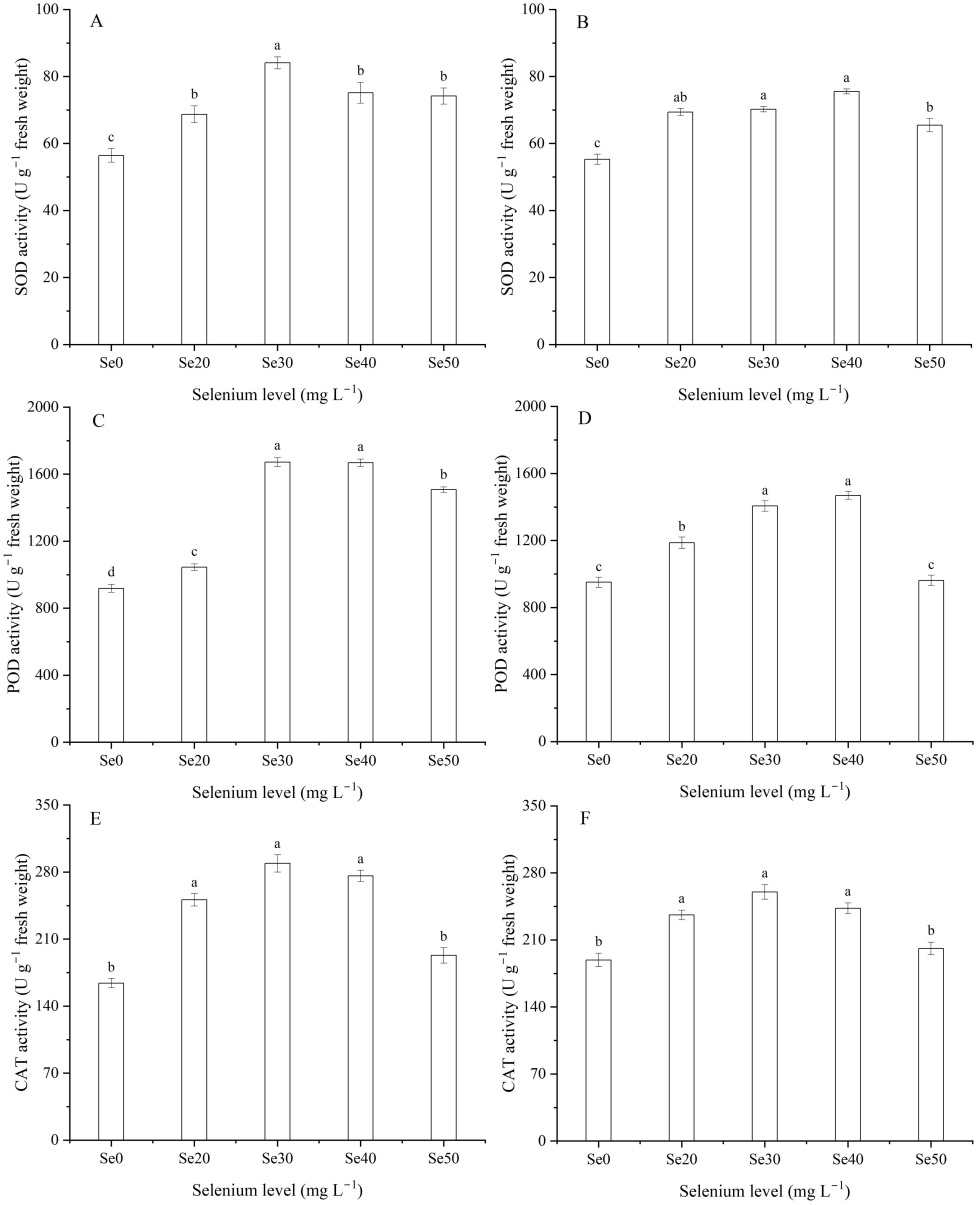
Effect of foliar selenium (Se) application on the activities of superoxide dismutase (SOD) **(A)** (2023) and **(B)** (2024), peroxidase (POD) **(C)** (2023) and **(D)** (2024), and catalase (CAT) **(E)** (2023) and **(F)** (2024) in flax leaves. Different letters indicate means in the same harvest that are significantly different at *P* = 0.05 according to Tukey’s test. Vertical bars represent standard errors (n=3).

Harvest significantly affected SOD activity (**Table 1**). The activity of SOD was 14.0% greater in 2023 than 2024. Superoxide dismutase, POD, and CAT activities varied significantly among the different levels of foliar Se fertilization (**Table 1**; **Figures 3A-F**). With the Se treatments, the SOD, POD, and CAT activities increased markedly. Compared to the control, in 2023, the SOD, POD, and CAT activities improved by 33.9, 60.5, and 53.8%, respectively; and in 2024, these activities increased by 27.0, 32.1, and 24.4%, respectively. As shown in the figure, the SOD, POD, and CAT activities initially increased and then decreased. The interaction between harvest and Se significantly influenced the SOD, POD, and CAT activities of flax (**Table 1**). The maximum activities of SOD and POD were observed at Se30 in 2023 and Se40 in 2024 (**Figures 3A-D**).

### Seed yield components and yield

In the present study, the number of capsules per plant and seed yield of flax significantly differed over the harvests (**Table 1**). The number of capsules per plant of flax was 28.9% greater in 2023 compared to 2024. Selenium level significantly impacted the number of capsules per plant in both harvests (**Tables 1**, **2**). Foliar Se application increased the number of capsules per plant by an average of 22.5 and 30.4% compared to the controls in 2023 and 2024, respectively. Harvest and Se interaction significantly impacted the number of capsules per plant and 1000-seed weight of flax (**Table 1**). The maximum of number of capsules per plant was observed at Se30 and Se40 in 2023 and 2024, respectively; representing increases of 32.4 and 42.5%, compared with the controls.

**Table 2:** Seed yield components of flax as affected by selenium rate.

Harvest	Selenium rate (mg L^−1^)	Number of capsules per plant	Number of seeds per capsule	1000-seed weight (g)
	0	–	–	–
	20	–	–	–
2022	30	–	–	–
	40	–	–	–
	50	–	–	–
	0	18.2 ± 0.21c^a^	6.65 ± 0.02a	6.58 ± 0.02a
	20	20.0 ± 0.15b	6.65 ± 0.04a	6.53 ± 0.02a
2023	30	24.1 ± 0.23a	6.68 ± 0.03a	6.64 ± 0.03a
	40	23.8 ± 0.18a	6.25 ± 0.06a	6.59 ± 0.03a
	50	21.3 ± 0.16b	632 ± 0.05a	6.46 ± 0.03a
	0	13.4 ± 0.21c	6.24 ± 0.05a	6.18 ± 0.03a
	20	16.6 ± 0.16b	6.36 ± 0.02a	6.31 ± 0.02a
2024	30	16.6 ± 0.18b	6.34 ± 0.04a	6.44 ± 0.03a
	40	19.1 ± 0.14a	6.74 ± 0.03a	6.51 ± 0.02a
	50	17.6 ± 0.12b	6.44 ± 0.05a	6.29 ± 0.02a

a Means in the same column and harvest followed by the same letter do not differ significantly according to the Tukey test (*P* = 0.05). – indicates data deficient, a confusion of samples occurred during the collecting process in 2022.

**Figure 4:**
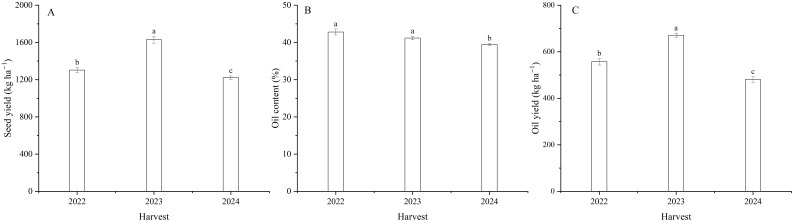
Effect of harvest on **(A)** seed yield, **(B)** oil content and **(C)** yield of flax. Different letters indicate means that are significantly different at *P* = 0.05 according to Tukey’s test. Vertical bars represent standard errors (n=3).

**Figure 5:**
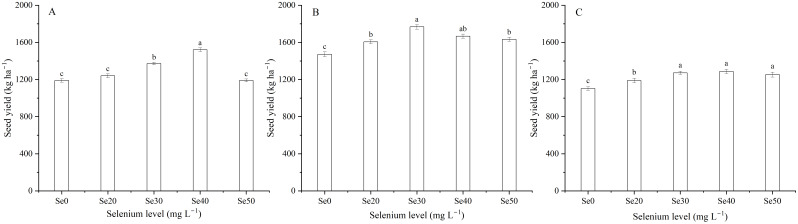
Effect of foliar selenium (Se) application on seed yield of flax in **(A)** 2022, **(B)** 2023, and **(C)** 2024. Different letters indicate means in the same harvest that are significantly different at *P* = 0.05 according to Tukey’s test. Vertical bars represent standard errors (n=3).

Harvest signally impacted the seed yield of flax (**Table 1**). The seed yield of flax averaged 1302.5 kg ha^–1^ in 2022, 1628.9 kg ha^–1^ in 2023, and 1220.6 kg ha^–1^ in 2024 (**Figure 4A**). With foliar Se treatments, seed yield increased by an average of 11.9, 13.3 and 13.3% in 2022, 2023, and 2024, respectively, compared to the controls. Moreover, there was no difference between the Se0, Se20, and Se50 treatments in 2022. Se30 and Se40 treatments in 2023, and Se30, Se40, and Se50 treatments in 2024. The seed yield was also affected by the interaction between harvest and Se. The highest seed yield increased by 27.8, 20.0, and 16.6%, respectively, compared to the controls. Moreover, Peak values were observed at Se40 in 2022, Se30 in 2023, and Se40 in 2024 (**Figures 5A-C**).

### Oil content and yield

**Figure 6:**
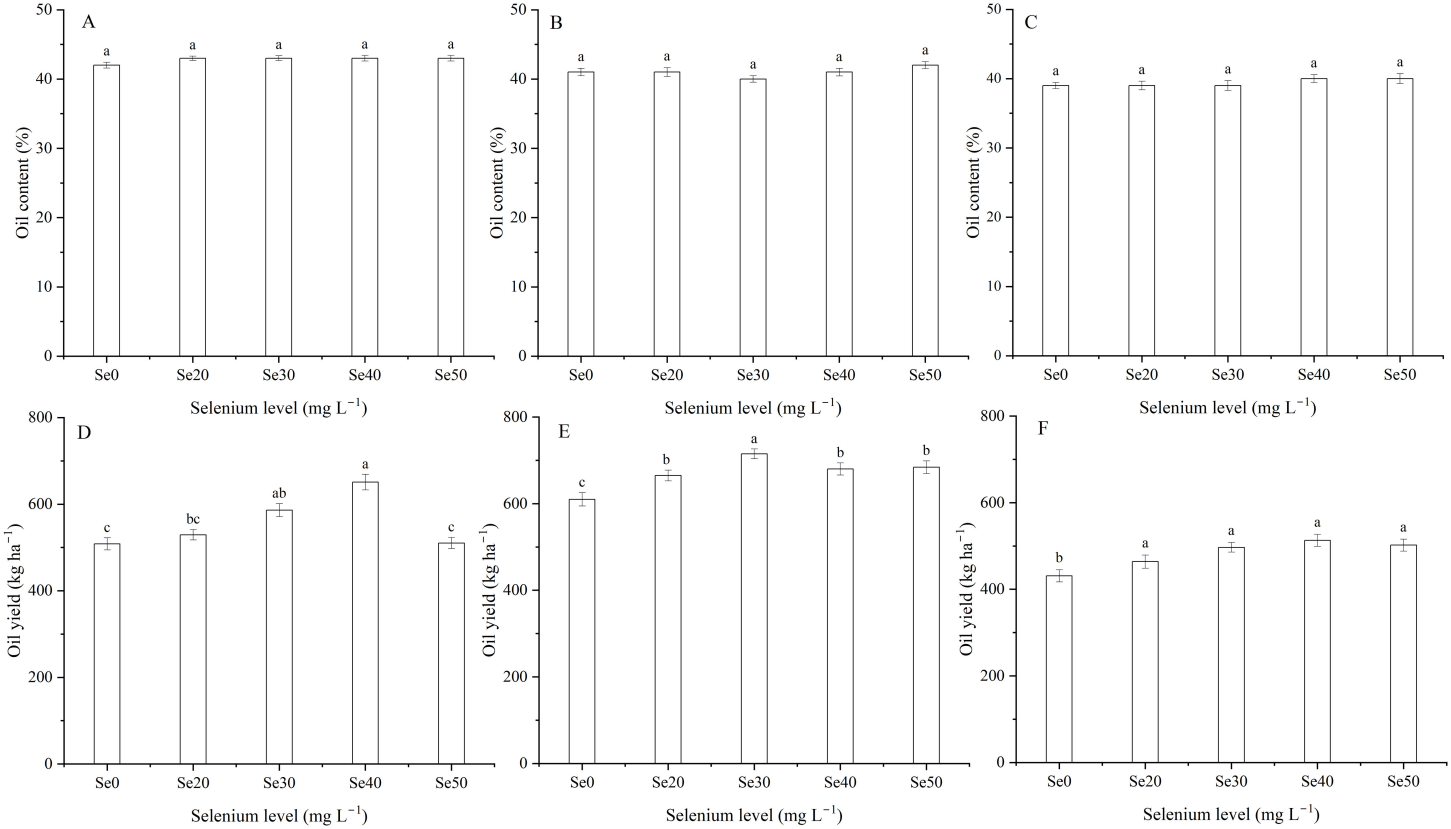
Effect of foliar selenium (Se) application on the oil content **(A)** (2022), **(B)** (2023), and **(C)** (2024) and yield **(D)** (2022), **(E)** (2023), and **(F)** (2024) of flax. Different letters indicate means in the same harvest that are significantly different at *P* = 0.05 according to Tukey’s test. Vertical bars represent standard errors (n=3).

Harvest dramatically impacted the oil content and yield of flax (**Table 1**; **Figures 4B, C**). The oil content was 8.5% higher in 2022 than 2024, and oil yield (Equation 1) was 39.3% greater in 2023 than 2024. Selenium had no significant effect on oil content (**Table 1**; **Figures 6A-C**). Oil yield differed significantly among different levels of Se fertilization (**Table 1**; **Figures 6D-F**). Compared to the controls, foliar Se treatments increased by an average of 12.0, 12.5, and 14.7% in 2022, 2023, and 2024, respectively. The interaction between harvest and Se affected the oil yield of flax (**Table 1**). The highest oil yield were calculated at Se40 in 2022, Se30 in 2023, and Se40 in 2024 (**Figures 6D-F**). Compared to the controls, these peak increased by 28.1, 17.3 and 19.0% in 2022, 2023, and 2024, respectively.

### Lignans concentration and yield

**Figure 7:**
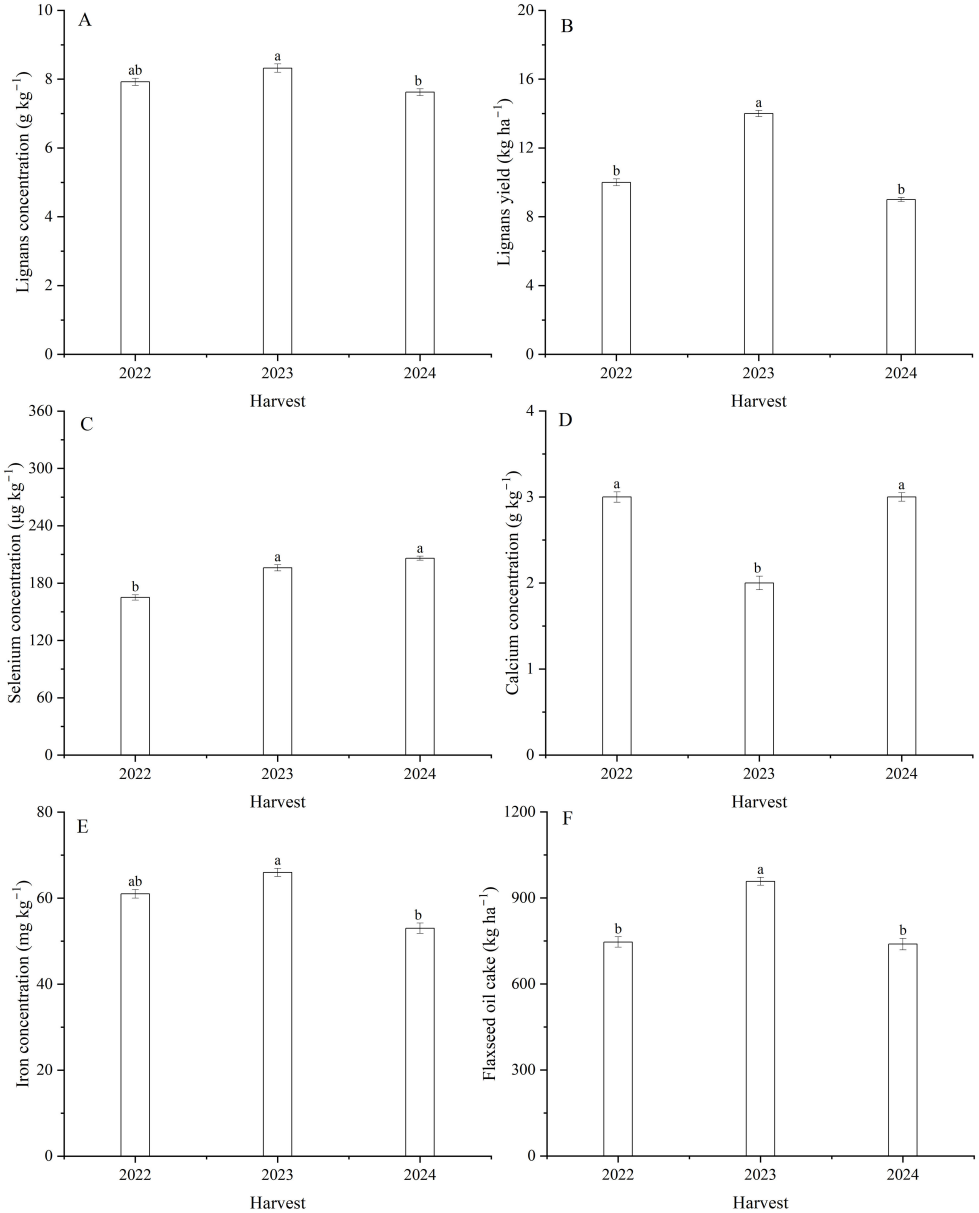
Effect of harvest on lignans **(A)** concentration and **(B)** yield, the concentrations of **(C)** Se, **(D)** Ca, and **(E)** Fe, as well as **(F)** flaxseed oil cake of flax. Different letters indicate means that are significantly different at *P* = 0.05 according to Tukey’s test. Vertical bars represent standard errors (n=3).

**Figure 8:**
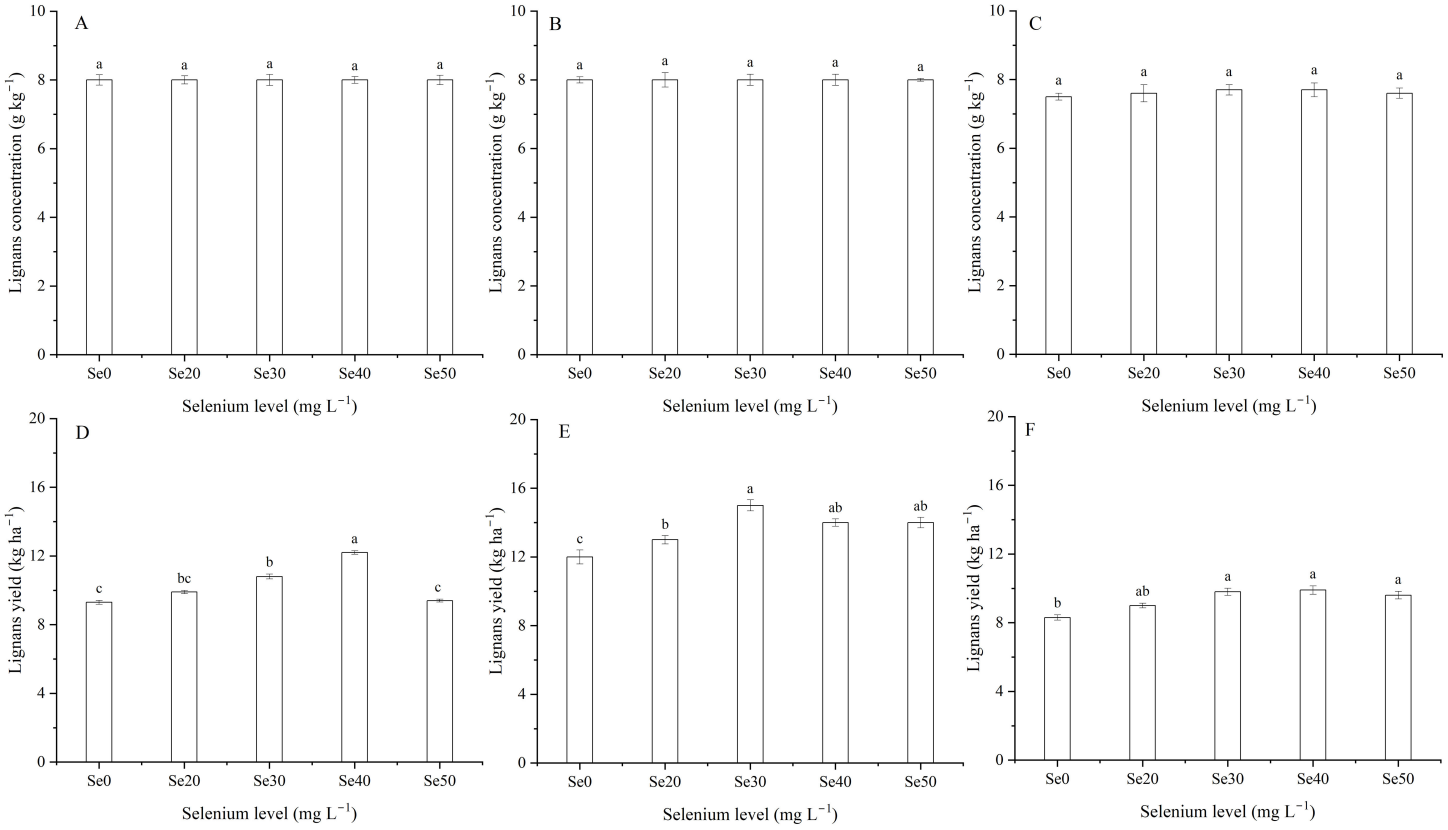
Effect of foliar selenium (Se) application on the lignans concentration **(A)** (2022), **(B)** (2023), and **(C)** (2024) and yield **(D)** (2022), **(E)** (2023), and **(F)** (2024) of flax. Different letters indicate means in the same harvest that are significantly different at *P* = 0.05 according to Tukey’s test. Vertical bars represent standard errors (n=3).

Harvest and the interaction between harvest and Se had a significant effect on lignans concentration in seeds (**Table 1**; **Figures 7A**, **8A–C**). The average lignans concentration was 7.9 g kg^–1^ in 2022, 8.3 g kg^–1^ in 2023, and 7.6 g kg^–1^ in 2024 (**Figure 7A**). Additionally, harvest markedly influenced the lignans yield of flax (Equation 2) (**Table 1**; **Figure 7B**). The average yield of lignans was 10.3 kg ha^–1^ in 2022, 13.6 kg ha^–1^ in 2023, and 9.3 kg ha^–1^ in 2024. The lignans yield was memorably impacted by foliar Se as well as the interaction between harvest and Se (**Table 1**). The maximum lignans yield was observed at Se40 in 2022, Se30 in 2023, and Se40 in 2024, respectively; compared to the controls, increasing by 31.0, 21.8, and 19.7%, respectively (**Figures 8D-F**). Furthermore, there were no significant differences between Se30, Se40, and Se50 treatments in 2023 and 2024, respectively.

### Fatty acid composition

Harvest dramatically impacted the oleic, linoleic, and linolenic acid contents in seeds (**Table 1**). The oleic content averaged 24.4, 25.0, and 26.5% in 2022, 2023, and 2024, respectively. The linoleic content averaged 13.8% in 2022, 15.0% in 2023, and 15.5% in 2024 (**Table 3**; **Supplementary Table 2**). The linolenic content, averaging 49.7% in 2022, 48.1% in 2023, and 45.9% in 2024 (**Table 3**; **Supplementary Table 2**). The harvest and Se interaction had a significantly influenced on oleic and linolenic acid contents (**Table 1**). The maximum oleic acid was observed at Se0 in 2022, Se50 in 2023, and Se30 in 2024.

**Table 3:** Fatty acid composition of flaxseed as affected by selenium rate in 2022, 2023, and 2024.

Harvest	Selenium rate (mg L^−1^)	Palmitic acid (%)	Stearic acid (%)	Oleic acid (%)	Linoleic (%)	Linolenic (%)
	0	5.5 ± 0.02a^a^	5.9 ± 0.01a	24.7 ± 0.16a	13.7 ± 0.08a	49.6 ± 0.26a
	20	5.6 ± 0.03a	6.0 ± 0.04a	24.3 ± 0.15a	13.8 ± 0.12a	49.8 ± 0.20a
2022	30	5.6 ± 0.02a	5.8 ± 0.02a	24.4 ± 0.15a	13.8 ± 0.09a	49.7 ± 0.18a
	40	5.5 ± 0.02a	6.0 ± 0.03a	24.3 ± 0.12a	13.8 ± 0.12a	49.5 ± 0.05a
	50	5.6 ± 0.03a	6.1 ± 0.03a	24.2 ± 0.14a	13.9 ± 0.15a	49.7 ± 0.24a
	0	5.2 ± 0.06a	6.0 ± 0.02a	24.8 ± 0.14a	15.4 ± 0.17a	48.4 ± 0.21a
	20	5.5 ± 0.03a	6.0 ± 0.06a	24.8 ± 0.17a	15.7 ± 0.07a	47.8 ± 0.15a
2023	30	5.5 ± 0.04a	5.9 ± 0.01a	25.2 ± 0.15a	14.7 ± 0.10a	48.0 ± 0.24a
	40	5.7 ± 0.02a	6.0 ± 0.07a	25.1 ± 0.09a	14.5 ± 0.14a	48.1 ± 0.17a
	50	5.8 ± 0.06a	5.9 ± 0.01a	25.3 ± 0.11a	14.8 ± 0.08a	48.1 ± 0.16a
	0	5.1 ± 0.03a	5.9 ± 0.02a	26.5 ± 0.15a	15.5 ± 0.08a	46.1 ± 0.36a
	20	5.7 ± 0.08a	5.9 ± 0.04a	26.2 ± 0.12a	15.7 ± 0.12a	45.6 ± 0.41a
2024	30	5.5 ± 0.02a	6.0 ± 0.09a	26.8 ± 0.14a	15.5 ± 0.11a	45.4 ± 0.23a
	40	5.5 ± 0.04a	6.0 ± 0.04a	26.2 ± 0.23a	15.6 ± 0.14a	46.1 ± 0.29a
	50	5.3 ± 0.04a	6.0 ± 0.05a	26.7 ± 0.12a	15.1 ± 0.07a	46.3 ± 0.22a

a Means in the same column and harvest followed by the same letter do not differ significantly according to Tukey test (*P* = 0.05).

### Selenium, iron, zinc, calcium, magnesium, and cadmium concentrations in seed

**Figure 9:**
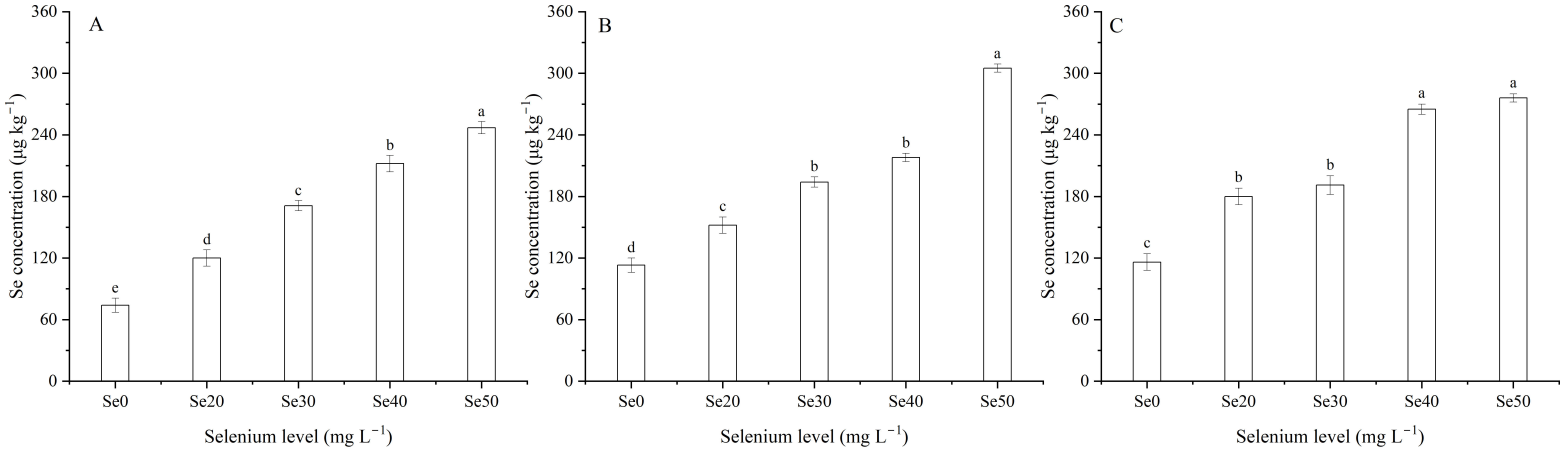
Effect of foliar selenium (Se) application on the Se concentration in flaxseeds in **(A)** (2022), **(B)** (2023), and **(C)** 2024. Different letters indicate means in the same harvest that are significantly different at *P* = 0.05 according to Tukey’s test. Vertical bars represent standard errors (n=3).

The concentrations of Se, Ca, and Fe in seeds were signally affected by harvest (**Table 1**; **Figures 7C-E**). The Se concentration in 2022, which was 16.1% lower than 2023 and 19.9% lower than 2024 (**Figure 7C**). The Ca concentration averaged 2.7 g kg^–1^ in 2022, 2.3 g kg^–1^ in 2023, and 2.7 g kg^–1^ in 2024 (**Figure 7D**). The Fe concentration was greater 24.6% in 2023 than 2024 (**Figure 7E**). Foliar Se application significantly impacted the Se, Fe, Zn, and Cd concentrations (**Tables 1**, **4**; **Figures 9A-C**). Compared to the zero Se, the Se, Fe, and Zn concentrations increased by an average of 152.9, 14.4, and 40.5% in 2022; 91.5, 10.6, and 37.3% in 2023, and 97.1, 14.3, and 62.0% in 2024, respectively. Conversely, the Cd concentration decreased by an average of 30.8% in 2022, 42.9% in 2023, and 33.4% in 2024.

**Table 4:** Effect of foliar selenium application on calcium (Ca), magnesium (Mg), iron (Fe), zinc (Zn), and cadmium (Cd) concentrations in seeds of flax in 2022, 2023, and 2024.

Harvest	Selenium rate (mg L^−1^)	Ca concentration (g kg^−1^)	Mg concentration (g kg^−1^)	Fe concentration (mg kg^−1^)	Zn concentration (mg kg^−1^)	Cd concentration (ųg kg^−1^)
	0	2.5 ± 0.08a^a^	4.0 ± 0.06a	54.8 ± 0.15c	27.9 ± 0.28b	44.0 ± 0.20a
	20	2.8 ± 0.13a	3.9 ± 0.05a	60.1 ± 0.20b	36.4 ± 0.54a	35.4 ± 0.15b
2022	30	2.7 ± 0.04a	3.8 ± 0.03a	64.3 ± 0.24a	39.5 ± 0.12a	36.2 ± 0.16b
	40	2.9 ± 0.08a	4.1 ± 0.05a	60.8 ± 0.19b	40.5 ± 0.23a	23.7 ± 0.10d
	50	2.7 ± 0.07a	3.9 ± 0.02a	65.4 ± 0.52a	40.4 ± 0.18a	28.9 ± 0.14c
	0	2.4 ± 0.06a	4.3 ± 0.05a	60.9 ± 1.72c	31.2 ± 1.01c	48.0 ± 1.26a
	20	2.9 ± 0.14a	5.1 ± 0.13a	65.9 ± 3.98b	40.0 ± 0.18b	33.9 ± 0.94b
2023	30	2.3 ± 0.07a	4.1 ± 0.09a	66.3 ± 3.63b	41.4 ± 0.19b	30.2 ± 0.78b
	40	2.1 ± 0.05a	3.8 ± 0.12a	69.5 ± 2.45a	45.6 ± 1.08a	20.6 ± 1.43d
	50	2.1 ± 0.09a	3.7 ± 0.18a	67.7 ± 3.20ab	44.3 ± 1.14a	25.0 ± 1.55c
	0	2.7 ± 0.07a	4.0 ± 0.20a	47.6 ± 3.04c	26.4 ± 1.06b	41.0 ± 0.86a
	20	2.8 ± 0.08a	4.2 ± 0.15a	50.1 ± 3.69c	42.1 ± 0.86a	32.1 ± 1.42b
2024	30	2.7 ± 0.09a	4.1 ± 0.07a	54.9 ± 2.58b	42.3 ± 0.92a	28.3 ± 1.17bc
	40	2.7 ± 0.08a	3.9 ± 0.11a	59.4 ± 3.12a	43.7 ± 1.03a	23.5 ± 1.04c
	50	2.7 ± 0.11a	3.8 ± 0.14a	53.2 ± 1.26b	43.0 ± 1.52a	25.4 ± 0.98c

a Means in the same column and harvest followed by the same letter do not differ significantly according to Tukey test (*P* = 0.05).

### Flaxseed oil cake

Flaxseed oil cake (Equation 3) showed significantly different between the harvests (**Table 1**; **Figure 7F**). The flaxseed oil cake was 29.6% greater in 2023 compared to 2024. The oil cake was impacted by Se application across harvests (**Table 1**; **Supplementary Figure 1**). The maximum oil cake yields increased by 27.7, 21.9, and 15.3% in 2022, 2023, and 2024, respectively, compared with the controls. Relative to the controls, the oil cake with Se treatments increased by an average of 11.8% in 2022, 13.8% in 2023, and 12.5% in 2024. There were no significant difference between the Se20, Se40, and Se50 treatments in 2023, nor between the Se20, Se30, Se40, and Se50 treatments in 2024 (**Supplementary Figure 1**).

## Discussion

### Effect of harvest

In the present study, harvest significantly impacted the proline and soluble sugar contents, SOD activity in leaves, the number of capsules per plant, seed yield, oil content and yield, lignans concentration and yield, the contents of oleic, linoleic, and linolenic acid, as well as the concentrations of Se, Ca, and Fe in flaxseeds and flaxseed oil cake. These variations may be correlated with differences in soil nutrients, precipitation, and air temperature during the vegetative, flowering, and seed-filling stages of flax (Xie et al., 2020, 2022). Čeh et al. (2020) also reported the seed yield of linseed was significantly affected by the harvest. In our experiment, seed yield in 2023 was 33.5% greater than of 2024. This difference can likely be attributed to:(i) lower rainfall during early flowering in 2024, which might have inhibited flower growth and development; (ii) lower rainfall and higher temperature during seed-filling in 2024, potentially decreasing the antioxidant defense system and photosynthesis, thereby affecting assimilate translocation during the seed-filling; and (iii) higher soil nutrients in 2023 than 2024. Fila et al. (2018) summarized that rainfall significantly influenced the seed yield of linseed. Moreover, higher post-flowering air temperature negatively affected on linseed seed yield (Fila et al., 2018). Similar effects were also observed by Čeh et al. (2020) in linseed. The increase in yield is a cumulative result of multiple yield components, such as 1000-seed weight, the number of seeds per capsule, and the number of capsules per plant, each responding to different environmental factors (Fila et al., 2018). Further research is required to explore the effects of these factors on the seed yield of flax.

In the current study, lignans concentration was affected by harvest, in line with our previous findings (Xie et al., 2022). The oil, oleic, linoleic, and linolenic acid contents were also impacted by harvest, which may be correlated with differences in temperature. As shown in **Figure 1**, higher temperature was recorded during the seed-filling period in 2024. This observation is supported by literature reporting that temperature can influence oil content (Green, 1986; Bernacchia et al., 2014; Fila et al., 2018) and fatty acid composition (Elferjani and Soolanayakanahally, 2018). It is now broadly recognized that linseed grown in cooler climates exhibits a higher oil content (Bernacchia et al., 2014). Elferjani and Soolanayakanahally (2018) found that high temperatures reduced the oil content and seed polyunsaturated fatty acids while increasing the monounsaturated fraction content in seed of canola. The results of this study were similar to those of Green (1986). This aligns with the results of Xie et al. (2020) in flax and Bellaloui et al. (2009) in soybean, who reported that linolenic acid content increased with lower average air temperatures during the seed-filling period. In a study of flax, Green (1986) noted that linoleic and linolenic acids significantly decreased, whereas oleic acid content increased as temperatures rose. This is in agreement with our study’s findings. Moreover, in our study, the oil and lignans yields were greater in 2023 than 2024, probably attributable to higher seed yield in 2023. These findings are consistent with previous studies on flax (Xie et al., 2020, 2022; Mirzaie et al., 2020).

Furthermore, the concentrations of Mg and Zn in flaxseed were not significantly affected by harvest. In line with our findings, previous studies have also shown that Zn concentrations in flaxseed did not display significant differences between the examined harvests (Xie et al., 2022). However, the Se, Ca, and Fe concentrations in flaxseeds exhibited significant differences between the three harvests, which is in agreement with the findings reported by Xie et al. (2022). In the present study, the Se and Ca concentrations were lower in 2023 compared to 2024, while the concentration of Fe was greater in 2023 than 2024. Those could be correlated with differences in soil nutrients and environment factors.

### Effect of foliar Se

In this study, foliar Se application significantly enhanced the contents of proline, and soluble sugar and the activities of SOD, POD, and CAT. It also increased the number of capsules per plant and the yields of seed, oil, ligans, and flaxseed oil cake. However, these traits reached their maximum levels and were subsequently inhibited when the selenium concentration increased beyond a certain threshold. These results confirmed the findings of Broadley et al. (2010); Pukacka et al. (2011); Pezzarossa et al. (2012) and Haghighi et al. (2019). They concluded that low dosages of Se exerts positive effects on growth enhancement, increased antioxidative capacity, reduced lipid peroxidation, improved yield and quality, as well as delayed ripening and senescence. Dai et al. (2019) also noted that Se utilization in Chinese cabbage (*Brassica rapa subsp. pekinensis*) significantly improved proline content, SOD, POD, and CAT activities, thereby enhancing its antioxidant system and biomass. This aligns with our current work, where foliar Se application improved the antioxidant system of flax by increasing proline and soluble sugar contents, as well as the activities of SOD, POD, and CAT, ultimately leading to an increase in the number of capsules per plant and seed yield. Shalaby et al. (2017) emphasized that Se-induced increases in antioxidant enzyme activity resulted in higher yields and nutritional quality in lettuce. Additionally, Sajedi et al. (2011) demonstrated that Se addition significantly enhanced antioxidant activity and corn (*Zea mays*) grain yield. In *Dracocephalum moldavica* L. Azimi et al. (2021) observed that Se treatments increased proline content, antioxidant enzyme activities, and essential oil constituents while decreasing MDA levels. Hussein et al. (2019) reported that foliar Se application enhanced antioxidant enzymes and soluble sugars, thereby strengthening the antioxidant defense system of groundnut. Consistent results were reported for lentil (Ekanayake et al., 2015), sorghum (Djanaguiraman et al., 2005, 2010), groundnut (Hussein et al., 2019), pakchoi (*Brassica rapa subsp. chinensis*) (Li et al., 2025), alfalfa (Bai et al., 2019), and other crops. Moreover, Logvinenko et al. (2022) in *Artemisia annua* L., Bai et al. (2019) in alfalfa, Wu et al. (2023) and Alves et al. (2020) in tomato, and Lin et al. (2012) in rice all found that foliar Se application decreased MDA content. Besides, Haghighi et al. (2019) indicated that POD and SOD activities increased, MDA content decreased, and flowers and fruit number improved with Se application at the high temperature, leading to increment in the yield of pepper. These results strongly supported the findings of the present study that foliar Se application increased POD and SOD activities, declined MDA content, enhanced its antioxidant system and antiaging physiology, improved the number of capsules per plant and seed yield of flax. Extensive literature has shown that Se application increased crop yield and Se content in seeds or edible portions, such as in lentil (Ekanayake et al., 2015), oilseed rape (Lyons et al., 2009), canola (Ahmad et al., 2021), camelina (Ahmad et al., 2021), safflower (Sher et al., 2022), and wheat (Nawaz et al., 2015; Liu et al., 2021). Moreover, Sher et al. (2022) indicated that Se treatments significantly improved the number of heads per plant, 1000-grain weight, grain yield and oil quality in safflower. Similarly, (Liu et al., 2021) found that Se application in wheat increased grain yield and its components (grain number and 1000-kernel weight) in wheat. Ahmad et al. (2021) concluded that foliar Se significantly increased the Se content in crop edible parts, which is consistent with our findings. However, Wang et al. (2013) reported no significant effect of foliar Se on maize grain yield, although Se concentration in grains was notably increased. This discrepancy may be attributed to differences in genotype. In this research, foliar Se did not affect the oil content or fatty acid composition in flaxseed. Conversely, in rapeseed, Davoudi et al. (2019) found that foliar Se application significantly increased the levels of oil, palmitic acid, oleic acid, and linoleic acid while markedly reducing linolenic acid content. This divergence probably stems from differences in genotype, environment, and their interactions.

Micronutrient supplementation has garnered significant attention due to its potential role in supporting immune function and overall human health. Iron, Zn, and Se deficiencies in humans are significant food-related issues on a global scale. Filek et al. (2019) documented that Se regulates the expression of genes involved in element transportation and accumulation in wheat. In the present study, the concentration of Se in seeds increased with increasing Se application levels, consistent with the conclusions of Wang et al. (2013). Similar results were observed in rice (Lu et al., 2024). Furthermore, the Se concentration in flaxseed (166.3~304.7 µg kg^–1^) with Se treatments aligns with the national industry standards for selenium content in grain and by-products (150~500 µg kg^–1^) (GH/T 1135-2024). Whereas, foliar Se applications did not significantly affect on the Ca, Mg, Fe, and Zn contents in maize (Wang et al., 2013). Liu et al. (2021) demonstrated that soil application of Se ore powder increased the concentrations of Zn, Fe, and Se in wheat grains. Similar results were reported for rice (Li et al., 2019) and peas (*Pisum sativum*) (Poblaciones and Rengel, 2017). In this study, the Fe and Zn concentrations in seeds initially increased but subsequently decreased with increasing Se levels, indicating low Se levels promote the accumulation of Fe and Zn, while high Se levels restrain their accumulation in flaxseeds. These phenomenon supported the results that Se fertilizer has dual effects on Fe uptake and translocation in plants (Gui et al., 2022). Evidence also suggests that Se within a certain range can enhance Zn accumulation in plants (Mangueze et al., 2018). Additionally, Dai et al. (2020) proposed that Se strongly increases Zn content in seeds of soybean. One probable reason is that the application of Se elevates the biosynthesis of SOD, thus indirectly increasing Zn uptake in plants (Ulhassan et al., 2019; Wu et al., 2020). This may be connected with the role of Zn as a cofactor in SOD synthesis (Georgiadou et al., 2018).

Cadmium (Cd) is the third most hazardous environmental contaminant and is uniquely recognized as a metal that poses health risks to both humans and animals (Ismael et al., 2019). Studies have shown that Se can reduce Cd content in the edible parts of various crops, including rice (Lin et al., 2012; Huang et al., 2018), tomato (Chi et al., 2017), pepper (Mozafariyan et al., 2014), and wheat (Khan et al., 2015). For instance, Huang et al. (2018) found that the addition of exogenous Se significantly increased Se content in rice grains by 4.25- and 2.39-fold while decreasing Cd level by 36.5 and 25.3%, respectively, compared to control treatments. In pakchoi, foliar Se application effectively reduced Cd concentration (Li et al., 2025). These phenomena are probably attributable to the fact that exogenous Se increased the levels of Cd bound to carbonate and iron-manganese oxides, thereby inhibiting Cd translocation from non-seed plant parts to seeds (Huang et al., 2018). Nevertheless, Se enhanced the Ca, Mg, and Zn concentration in rice (Feng et al., 2013). In the present study, the Ca and Mg concentrations did not significantly impacted by foliar Se application. The differences observed among crops can be mainly attributed to variations in genotype and climate conditions. Additionally, flaxseed oil cake reached peak at Se30 and then decreased. Further research is required to fully explore the benefits of Se on flax production.

## Conclusion

Flax has emerged as one of the most important prospective sources for food, fiber, and industrial applications due to its superior quality. Foliar Se application is an efficient management practice in field production. In this experiment, we investigated the effects of foliar Se application on physiology and biochemistry, yields and quality of flax. Result demonstrated that appropriate foliar Se application significantly enhanced yields and quality of flax. In summary, foliar application of 30 mg L^–1^ Se effectively decreased MDA content, improved proline and soluble sugar contents and SOD, POD, and CAT activities, and enhanced antioxidant defense system and antiaging physiology of flax. This approach also promoted absorption and accumulation to Se, Fe, and Zn in seeds, ultimately leading to enhance yields and quality (**Supplementary Figure 2**). This study represents the first comprehensive report on the effects of foliar Se on leaf physiology and biochemistry, productivity, and quality, including the Se, Ca, Mg, Fe, Zn, and Cd content in seeds of flax. In conclusion, appropriate foliar Se application can be a promising strategy to enhance flax productivity and achieve biofortification with Se, Fe, and Zn.

## Data Availability

The original contributions presented in the study are included in the article/**Supplementary Material**. Further inquiries can be directed to the corresponding authors.

## Supplementary material

Supplementary Figure 1 Effect of foliar selenium (Se) application on flaxseed oil cake in **(A)** (2022), **(B)** (2023), and **(C)** 2024. Different letters indicate means in the same harvest that are significantly different at *P* = 0.05 according to Tukey’s test. Vertical bars represent standard errors (n=3).

Supplementary Table 1 Soil chemical characteristics for the 0- to 30 cm depth at Qinwangchuan in 2022, 2023, and 2024.

Supplementary Table 2 Effect of harvest on fatty acid composition of flaxseed. a Means in the same column followed by the same letter do not differ significantly according to the Tukey test (*P* = 0.05).
