# Supplementary figures and images for: Effect of foliar spray selenium on antioxidant defense system, yields, fatty acid composition, and mineral concentrations in flax (Linum usitatissimum L.)
Source: Front Plant Sci. 2025 Jun 13;16:1600173. doi: 10.3389/fpls.2025.1600173 (PMC12202544; doi:10.3389/fpls.2025.1600173)

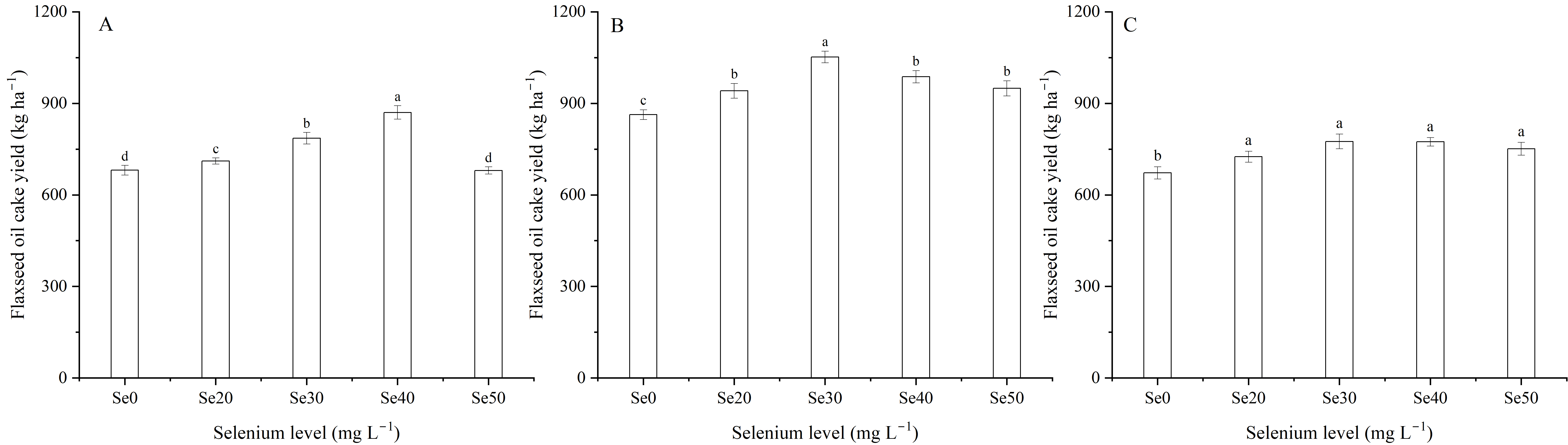

Supplement: Supplementary Figure 1 — Effect of foliar selenium (Se) application on flaxseed oil cake in (A) (2022), (B) (2023), and (C) 2024. Different letters indicate means in the same harvest that are significantly different at P = 0.05 according to Tukey’s test. Vertical bars represent standard errors (n=3). [file Image1.jpeg]

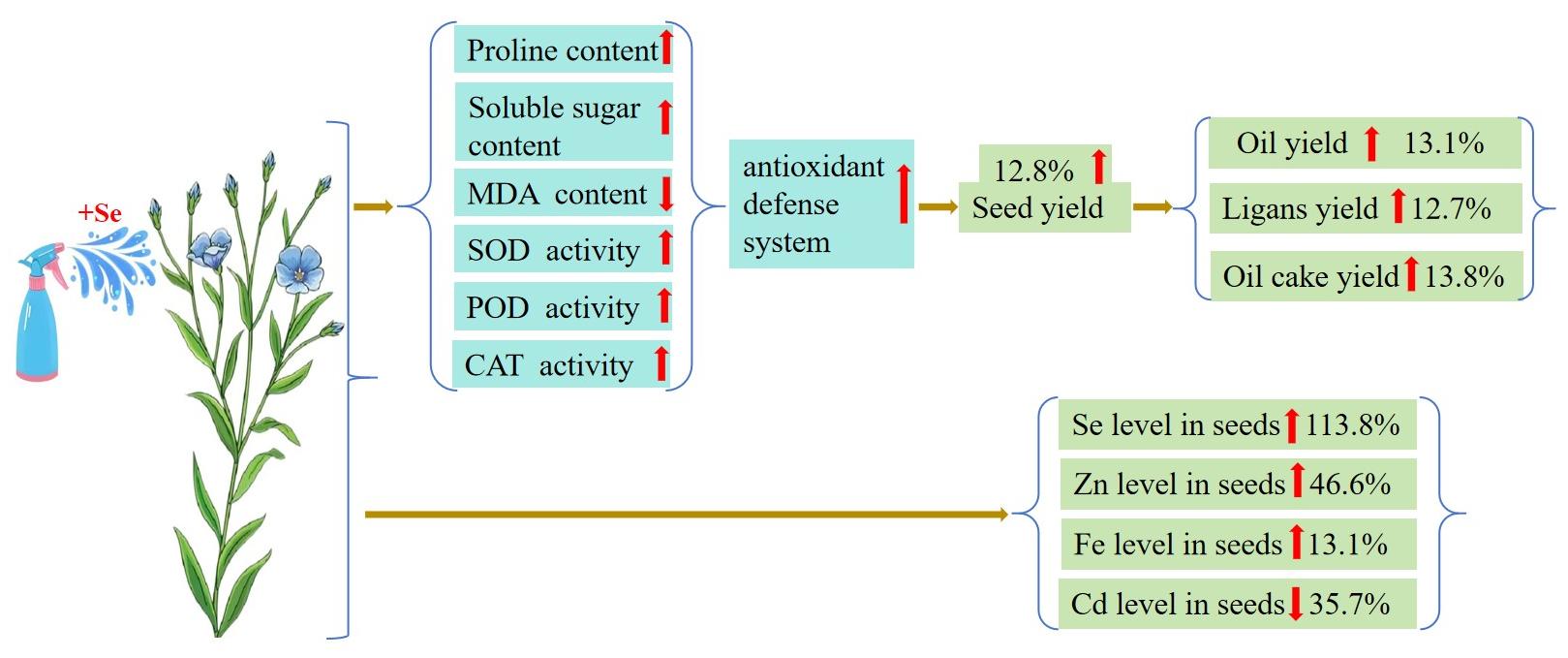

Supplement: Supplementary file 4 [file Image2.jpeg]
