# Supplementary material for: Effect of foliar spray selenium on antioxidant defense system, yields, fatty acid composition, and mineral concentrations in flax (Linum usitatissimum L.)
Source: Front Plant Sci. 2025 Jun 13;16:1600173. doi: 10.3389/fpls.2025.1600173 (PMC12202544; doi:10.3389/fpls.2025.1600173)
Supplement: Supplementary Table 1 — Soil chemical characteristics for the 0- to 30 cm depth at Qinwangchuan in 2022, 2023, and 2024. [file Table1.docx]

Supplementary Table 1 Soil chemical characteristics for the 0- to 30 cm depth at

Qinwangchuan in 2022, 2023, and 2024.

| Harvest | 2022 | 2023 | 2024 |
| --- | --- | --- | --- |
| Organic matter (g kg^–1^) | 11.8 | 13.2 | 10.9 |
| Alkali-hydrolyzable nitrogen (mg kg^–1^) | 57.5 | 63.4 | 51.7 |
| Available phosphorus (mg kg^–1^) | 25.4 | 29.5 | 23.5 |
| Available potassium (mg kg^–1^) | 221.1 | 244.1 | 181.5 |
| pH | 8.3 | 8.2 | 8.5 |
| Total selenium (mg kg^–1^) | 1.0 | 1.2 | 1.6 |
