# Supplementary material for: Effect of foliar spray selenium on antioxidant defense system, yields, fatty acid composition, and mineral concentrations in flax (Linum usitatissimum L.)
Source: Front Plant Sci. 2025 Jun 13;16:1600173. doi: 10.3389/fpls.2025.1600173 (PMC12202544; doi:10.3389/fpls.2025.1600173)
Supplement: Supplementary Table 2 — Effect of harvest on fatty acid composition of flaxseed. a Means in the same column followed by the same letter do not differ significantly according to the Tukey test (P = 0.05). [file Table2.docx]

Supplementary Table 2 Effect of harvest on fatty acid composition of flaxseed.

| Year | Palmitic acid  (%) | Stearic acid  (%) | Oleic acid  (%) | Linoleic  (%) | Linolenic  (%) |
| --- | --- | --- | --- | --- | --- |
| 2022 | 5.56±0.03a^a^ | 5.96±0.02a | 24.38±0.08b | 13.80±0.05b | 49.66±0.18a |
| 2023 | 5.54±0.04a | 5.96±0.03a | 25.04±0.06b | 15.02±0.04a | 48.08±0.20b |
| 2024 | 5.42±0.01a | 5.96±0.02a | 26.48±0.10a | 15.48±0.04a | 45.90±0.15c |

a Means in the same column followed by the same letter do not differ significantly according to the Tukey test (*P* = 0.05).
